# Long-term intraocular pressure-lowering efficacy and safety of ripasudil-brimonidine fixed-dose combination for glaucoma and ocular hypertension: a multicentre, open-label, phase 3 study

**DOI:** 10.1007/s00417-024-06388-y

**Published:** 2024-03-02

**Authors:** Hidenobu Tanihara, Tetsuya Yamamoto, Makoto Aihara, Noriko Koizumi, Atsuki Fukushima, Koji Kawakita, Satoshi Kojima, Toka Nakamura, Hideki Suganami, K-232 Clinical Study Group, Yoshitsugu Tagawa, Hiroki Watanabe, Kiyoshi Shimizu, Miki Iwasaki, Sakae Matsuzaki, Hiroko Ueda, Ryoko Okayama, Osamu Matsuoka, Setsuko Hashida, Sachi Amaki Kobayashi, Motohiro Kiyosawa, Yuko Asai, Toru Nakajima, Yuzuru Yoshimura, Takao Sakai, Ryoji Nomura, Satoshi Inoue, Ken Hayashi, Junko Watanabe, Hidehito Kawabata, Tomoyuki Muramatsu, Mikki Arai, Masayoshi Migita

**Affiliations:** 1 Department of Ophthalmology, Biei Municipal Hospital, 3-8-35 Naka-machi, Biei Town, Kamikawa-gun, Hokkaido, 071-0207 Japan; 2 Prof. Kazuo Iwata Memorial Kaijin Glaucoma Center, Kaiya Eye Clinic, Shizuoka, Japan; 3 Department of Ophthalmology, The University of Tokyo, Tokyo, Japan; 4 Department of Biomedical Engineering, Faculty of Life and Medical Sciences, Doshisha University, Kyoto, Japan; 5 Department of Ophthalmology, Tsukazaki Hospital, Hyogo, Japan; 6 Pharmaceutical Clinical Development Management Department, Kowa Company, Ltd., Tokyo, Japan; 7 Medical Affairs Department, Kowa Company, Ltd., Tokyo, Japan; 8 Data Science Center, Kowa Company, Ltd., Tokyo, Japan

**Keywords:** Glaucoma, Intraocular pressure, Long-term treatment, Ocular hypertension, Ripasudil-brimonidine fixed-dose combination

## Abstract

**Purpose:**

To evaluate the long-term efficacy and safety of ripasudil-brimonidine fixed-dose combination (RBFC), a new intraocular pressure (IOP)-lowering medication for glaucoma and ocular hypertension (OHT).

**Methods:**

This prospective, multicentre (23 sites in Japan), open-label study enrolled patients with primary open-angle glaucoma (POAG), OHT or exfoliative glaucoma and assigned them to one of four combination therapy cohorts, based on previous treatment(s) received: prostaglandin (PG) analogue (Cohort 1); PG analogue and beta-adrenoceptor blocker (β-blocker) (Cohort 2); PG analogue, β-blocker and carbonic anhydrase inhibitor (Cohort 3); or other/no treatment (Cohort 4). After a ≥ 4-week screening period, eligible patients received twice-daily RBFC for 52 weeks in addition to the treatments they were already receiving. Efficacy was assessed by change in IOP from baseline through week 52. Adverse events and adverse drug reactions (ADRs) were monitored throughout.

**Results:**

In total, 179 patients from Cohort 1 (*n* = 48), Cohort 2 (*n* = 44), Cohort 3 (*n* = 41) and Cohort 4 (*n* = 46) entered the RBFC treatment period. For all cohorts, mean IOP was significantly reduced at 11:00 (2 h after instillation of RBFC) through week 52 with the changes from baseline at week 52 of − 2.7 to − 4.1 mmHg across cohorts; all *p* < 0.001. Common ADRs were conjunctival hyperaemia (58%), allergic conjunctivitis (18%) and blepharitis (17%), most of which were mild in severity.

**Conclusion:**

These data demonstrated the long-term efficacy and safety of RBFC, both alone and in combination with other anti-glaucoma agents. RBFC may offer a new treatment option for the long-term management of glaucoma and OHT.

**Trial registration:**

Japan Registry of Clinical Trials Identifier: jRCT2080225063.

**Date of registration:**

17 February 2020.

**Supplementary Information:**

The online version contains supplementary material available at 10.1007/s00417-024-06388-y.


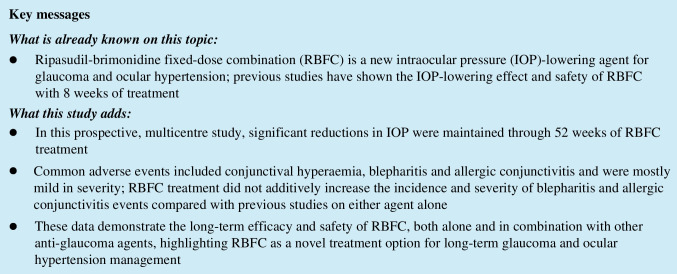


## Introduction

The most evidence-based and reliable treatment approach for glaucoma is a reduction in intraocular pressure (IOP), which is typically achieved with topical ocular hypotensive agents [1–3]. Studies have shown that long-term medication adherence is essential for delaying visual field progression in patients with both early- and advanced-stage glaucoma [4, 5]. Monotherapy is recommended at treatment initiation; if the target IOP is not reached with a single agent, combination therapy with two or more agents should be considered [1–3]. However, combination therapy is typically associated with poor medication adherence, in part due to increased treatment burden and regimen complexity (i.e. polypharmacy with multiple agents, each with different dosing schedules and instillation intervals) [6–8]. To promote adherence in the long-term management of glaucoma, fixed-dose combination therapies have been regarded as a useful solution to this problem [9, 10].

Ripasudil-brimonidine fixed-dose combination (RBFC; also known as K-232) is a new ocular hypotensive medication that combines ripasudil hydrochloride hydrate (ripasudil; a Rho-associated coiled-coil containing protein kinase [ROCK] inhibitor) with brimonidine tartrate (brimonidine; an alpha-2-adrenoceptor agonist [α_2_-agonist]). Current fixed-dose combinations for treatment of glaucoma and ocular hypertension (OHT) commonly include one or two prostaglandin (PG) analogues, beta-adrenoceptor blockers (β-blockers) or carbonic anhydrase inhibitors (CAIs). Prescription of multiple fixed-dose combinations with overlapping active ingredients is not recommended [3]. From this point of view RBFC’s new formulation, which does not contain these more common agents, represents a novel medical treatment option for glaucoma and OHT, in combination with existing IOP-lowering medications.

Two phase 3 clinical studies have demonstrated the IOP-lowering efficacy and safety of RBFC over 8 weeks in patients with primary open-angle glaucoma (POAG) or OHT [11]. In these studies, patients receiving RBFC had significantly greater reductions in IOP than those receiving ripasudil or brimonidine alone [11]. However, it is important to evaluate the long-term IOP-lowering effects and safety profile of RBFC for the management of glaucoma and OHT, because studies have shown higher incidences of allergic responses and resultant occurrence of blepharitis and conjunctivitis in eyes with treated with ripasudil and/or brimonidine [12–16]. Herein, we report the results of a multicentre, open-label, phase 3 study in patients with glaucoma or OHT designed to evaluate the long-term (52-week) efficacy and safety of RBFC, both alone and as a concomitant medication.

## Methods

### Study design

This was a prospective, multicentre, open-label, long-term study conducted at 23 clinical sites in Japan between 19 February 2020 and 5 November 2021. The study was approved by the Institutional Review Boards of participating sites prior to commencement and was conducted in compliance with the Declaration of Helsinki and Ministry of Health and Welfare Ordinance on Good Clinical Practice. The study is registered with the Japan Registry of Clinical Trials (jRCT identifier: jRCT2080225063). All patients provided written informed consent prior to study participation.

### Study population

Eligible patients for the screening period were adults aged ≥ 20 years with POAG, OHT, exfoliative glaucoma (EXG) or pigmentary glaucoma. Patients were reassessed at 09:00 on day 1 of the treatment period (before the initiation of RBFC). To be eligible for the treatment period, patients had to have an IOP (as measured by Goldmann applanation tonometry) that was ≥ 15 mmHg in at least one eye diagnosed with POAG, OHT, EXG or pigmentary glaucoma, and < 35 mmHg in both eyes. Main exclusion criteria were patients with narrow angle closure in either eye (Shaffer classification grade 0–2), best-corrected visual acuity (BCVA) of 20/70 or worse in either eye, and previous ocular surgery or laser treatment in either eye (except for retinal photocoagulation or yttrium aluminium garnet [YAG] laser capsulotomy ≥ 90 days before screening, eyelid surgery ≥ 120 days before screening or cataract surgery ≥ 1 year before screening). Participants were prohibited from receiving other IOP-lowering agents (except for prespecified concomitant agents), ocular surgery or laser treatment, or from using contact lenses throughout the study period. Full inclusion and exclusion criteria are provided in Supplementary Table S1 in Online Resource 1.

### Intervention and follow-up

After providing consent, patients entered a screening period of ≥ 4 weeks, up to a maximum of 6 weeks, followed by a 52-week treatment period (Supplementary Fig. S1; Online Resource 1). At the start of the screening period, study investigators assigned patients to one of four cohorts based on the patients’ treatment prior to study entry. Patients received a PG analogue (Cohort 1), PG analogue and β-blocker (Cohort 2), PG analogue, β-blocker and CAI (Cohort 3), or other/no treatment (Cohort 4) during the screening period. In addition, patients in all cohorts could receive ripasudil 0.4% or brimonidine 0.1% during the screening period, per investigator discretion. All agents were administered in line with their prescribing information. In all cohorts, thrice-daily drugs were instilled at 09:00, 15:00 and 21:00, twice-daily drugs were instilled at 09:00 and 21:00, and once-daily drugs were instilled at either 09:00 or 21:00.

Agents outside of the assigned cohorts and/or agents that the investigators chose not to use were washed out during the screening period. A ≥ 4-week washout applied to patients previously treated with a PG analogue, β-blocker, alpha-1/beta-adrenoceptor blocker (α_1_β-blocker), alpha-1-adrenoceptor blocker (α_1_-blocker), α_2_-agonist, ROCK inhibitor or prostanoid EP2 receptor agonist, or any combination of these glaucoma agents including fixed-dose combination products. A ≥ 2-week washout applied to patients previously treated with any other glaucoma agent. For patients in Cohort 4 who did not receive any type of combination therapy, including ripasudil 0.4% or brimonidine 0.1%, during the screening period, the treatment period could start ≥ 1 day after the start of the screening period, provided that any applicable washout criteria were met.

On day 1 of the treatment period, patients who satisfied IOP eligibility criteria at 09:00 received RBFC (ophthalmic solution containing ripasudil 0.4% and brimonidine 0.1%; 1 drop per eye) on top of the concomitant treatment they received during the screening period (or RBFC alone in Cohort 4 patients who received no treatment). Ripasudil 0.4% or brimonidine 0.1% were switched to RBFC if either agent had been used during the screening period. Thereafter, RBFC was instilled twice daily at around 09:00 and 21:00 through week 52. Patients attended study visits at the start of the screening period, day 1 of the treatment period, week 2 and then every 4 weeks from week 4 through week 52.

At each visit, IOP in both eyes was measured by Goldmann applanation tonometry at 09:00 (before instillation) and 11:00 (2 h after instillation). Other ocular assessments (slit lamp microscopy, BCVA and visual field tests, fundus examination, ultrasound pachymetry for corneal thickness, non-contact specular microscopy for corneal endothelial cell morphology and density), clinical examinations (blood pressure, heart rate) and laboratory tests (haematology, clinical chemistry, urinalysis) were performed at prespecified study visits. Adverse events (AEs) and adverse drug reactions (ADRs) were monitored throughout the study period.

### Outcome measures

The efficacy outcome was change in IOP from baseline through week 52. Baseline IOP was measured at two time points on treatment day 1: at 9:00 and 11:00, both prior to first instillation of RBFC. Change in IOP from baseline was measured as the difference between: (1) baseline IOP measured at 09:00 on day 1 and at 09:00 (before instillation) on each study visit; and (2) baseline IOP at 11:00 on day 1 and at 11:00 (2 h after instillation) on each study visit. One eye per patient that satisfied IOP eligibility criteria at 09:00 on day 1 of the treatment period (before the initiation of RBFC) was selected as the study eye for the efficacy analysis. If both eyes were eligible, the eye with higher IOP was selected; if IOP was the same in both eyes, the right eye was selected.

Safety was evaluated through the incidence and severity of AEs and ADRs, ocular assessments (excluding IOP), clinical examinations and laboratory tests throughout the treatment period. AEs and ADRs were coded using the Medical Dictionary for Regulatory Activities Terminology thesaurus terms (MedDRA, version 24.0), and the severity of events were classified as mild (i.e. does not affect daily activities), moderate (i.e. does affect some daily activities) or severe (i.e., unable to do normal daily activities). Corneal endothelial cell morphology was evaluated using non-contact specular microscopy and classified into one of four grades or as ‘undeterminable’: Grade 0 = normal endothelial cell morphology (cells appear as white polygons with black borders) with no findings similar to guttae (corneal guttae findings are black irregularly shaped cells with white borders); Grade 1 = many endothelial cells have clear borders, but ≥ 10% are partially blackened out with findings similar to guttae; Grade 2 = endothelial cell borders are indistinct, with many having findings similar to guttae; Grade 3 = endothelial cell borders cannot be identified; undeterminable = not applicable to any of Grade 0–3 [17].

### Statistical analysis

A sample size of 160 patients (i.e. 40 patients per cohort) was estimated to ensure that the safety of RBFC could be evaluated in ≥ 100 patients for 1 year, in line with International Council for Harmonisation E1 Guidelines. Efficacy and safety analyses were based on the full analysis set. In the efficacy analyses, IOP and change in IOP from baseline over time were summarised using means and standard deviation (SD) for each cohort and the total study population. Subgroup analyses were performed to assess change in IOP by age (< 65 or ≥ 65 years), sex, diagnosis (POAG, OHT or EXG), baseline IOP (< 17.5 or ≥ 17.5 mmHg) and concomitant agent (PG analogue, β-blocker and CAI). Change in IOP from baseline at each point was analysed using a one-sample t-test. The two-sided significance level was 0.05 and the two-sided confidence interval (CI) was 95%.

In the safety analyses, the incidence and severity of AEs and ADRs through study end were assessed using descriptive summaries for each cohort and the total study population. All statistical analyses were performed by Kowa Company, Ltd. using SAS version 9.4 (SAS Institute, Cary, NC, USA).

## Results

### Study population

In total, 200 patients with POAG, OHT or EXG provided consent and entered the screening period (Supplementary Fig. S2; Online Resource 1). Among those, 21 patients discontinued the study during screening, four patients withdrew consent, two withdrew due to an AE, one withdrew due to physician decision and 14 patients (67%) failed to meet criteria on the first treatment day. The remaining 179 patients entered the treatment period and received ≥ 1 dose of RBFC. Of these, 48 patients were in Cohort 1 (PG analogue), 44 in Cohort 2 (PG analogue and β-blocker), 41 in Cohort 3 (PG analogue, β-blocker and CAI) and 46 in Cohort 4 (other/no treatment). These 179 patients made up the full analysis set for the efficacy and safety analyses (Supplementary Fig. S2; Online Resource 1).

Overall, 141 patients (79%) completed the 52-week treatment period. Most patients who did not complete the RBFC treatment period discontinued due to AEs or reasons related to AEs (36/38 patients; 95%), with no difference in the proportion of patients who discontinued treatment between cohorts (Supplementary Fig. S2; Online Resource 1).

Baseline patient characteristics in the full analysis set were similar across cohorts (Table 1). Overall, the majority of patients were diagnosed with POAG (84%) or OHT (15%); only two patients (1%) had EXG, and none had pigmentary glaucoma. Most patients (94%) had previously received IOP-lowering therapy for the last 2 years, and during the screening period, only nine (5%) and 13 (7%) patients received ripasudil 0.4% and brimonidine 0.1%, respectively.

**Table 1:** Baseline patient demographics and clinical characteristics

	Cohort 1 (*n* = 48)	Cohort 2 (*n* = 44)	Cohort 3 (*n* = 41)	Cohort 4^a^ (*n* = 46)	Total (*n* = 179)
Age (years)	59.9 ± 13.8	65.3 ± 9.5	63.5 ± 10.0	65.2 ± 9.1	63.4 ± 11.0
Sex					
Male	25 (52.1)	26 (59.1)	26 (63.4)	22 (47.8)	99 (55.3)
Female	23 (47.9)	18 (40.9)	15 (36.6)	24 (52.2)	80 (44.7)
Diagnosis					
POAG	42 (87.5)	34 (77.3)	39 (95.1)	36 (78.3)	151 (84.4)
OHT	6 (12.5)	8 (18.2)	2 (4.9)	10 (21.7)	26 (14.5)
EXG	0	2 (4.5)	0	0	2 (1.1)
Pigmentary glaucoma	0	0	0	0	0
Baseline IOP^b^ (mmHg)					
At 09:00	17.5 ± 1.7	18.8 ± 3.5	17.6 ± 1.9	19.1 ± 3.3	18.2 ± 2.8
At 11:00	16.5 ± 2.0	17.5 ± 3.3	16.0 ± 2.1	17.4 ± 3.0	16.8 ± 2.7
Previous treatment^c^					
Yes	47 (97.9)	43 (97.7)	41 (100.0)	37 (80.4)	168 (93.9)
No	1 (2.1)	1 (2.3)	0	9 (19.6)	11 (6.1)
Received ripasudil or brimonidine during screening period					
Ripasudil	0	0	6 (14.6)	3 (6.5)	9 (5.0)
Brimonidine	2 (4.2)	2 (4.5)	4 (9.8)	5 (10.9)	13 (7.3)
None	46 (95.8)	42 (95.5)	31 (75.6)	38 (82.6)	157 (87.7)
History of allergy^d^					
Yes	26 (54.2)	25 (56.8)	16 (39.0)	22 (47.8)	89 (49.7)
No	22 (45.8)	19 (43.2)	25 (61.0)	24 (52.2)	90 (50.3)

αβ*-blocker* Alpha-1/beta-adrenoceptor blocker; β*-blocker* Beta-adrenoceptor blocker; *CAI* Carbonic anhydrase inhibitor; *EP2* Prostanoid EP2 receptor; *EXG* Exfoliative glaucoma; *IOP* Intraocular pressure; *OHT* Ocular hypertension; *PG* Prostaglandin; *POAG* Primary open-angle glaucoma; *SD* Standard deviation

Values are the number of patients (%) or mean ± SD

^a^Concomitant agents received by patients in Cohort 4 were: β-blocker and CAI (*n* = 8), β-blocker (*n* = 7), EP2 agonist (*n* = 2), CAI (*n* = 1), PG analogue and CAI (*n* = 1), β-blocker, CAI and EP2 agonist (*n* = 1), β-blocker and αβ-blocker (*n* = 1); 25 patients received no treatment

^b^Baseline IOP was measured at 09:00 and 11:00 on day 1 of the treatment period before the initiation of RBFC

^c^Previous treatment ≤ 2 years before the start of the screening period

^d^Any incident of allergic disease within 1 year or allergy from any cause including pollen, food and medication

After continued treatment with therapies used prior to study entry (or no treatment; as part of Cohort 4) during the screening period, the mean ± SD baseline IOP in the study eye was 18.2 ± 2.8 mmHg at 09:00 and 16.8 ± 2.7 mmHg at 11:00 on day 1 of the treatment period.

### IOP-lowering effect of RBFC

**Fig. 1:**
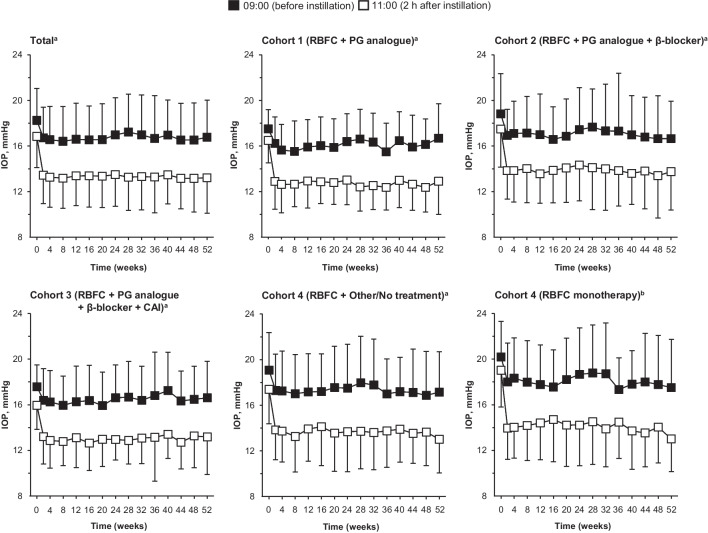
Mean ± SD intraocular pressure at 09:00 (before instillation) and 11:00 (2 h after instillation) from baseline through week 52 of the treatment period. β*-blocker* Beta-adrenoceptor blocker, *CAI* Carbonic anhydrase inhibitor, *IOP* Intraocular pressure, *PG* Prostaglandin, *RBFC* Ripasudil-brimonidine fixed-dose combination, *SD* Standard deviation. ^a^Total, *n* = 179; Cohort 1, *n* = 48; Cohort 2, *n* = 44; Cohort 3, *n* = 41; Cohort 4, *n* = 46. For total study population and all cohorts, mean IOP was significantly reduced from baseline at each time point (*p* < 0.001 and *p* < 0.05, respectively), except for IOP measurements taken at 09:00 on week 36 and week 40 in Cohort 3. ^b^Subgroup of patients in Cohort 4 who did not receive any combination therapy, including ripasudil 0.4% or brimonidine 0.1%, during the screening and treatment periods (*n* = 21). In this subgroup, mean IOP was significantly reduced from baseline at each time point (*p* < 0.05), except for IOP measurements taken at 09:00 on week 28 and week 32

For all cohorts, the mean IOP at 09:00 and 11:00 in the study eye was reduced after instillation of RBFC, and this IOP-lowering effect was maintained from week 2 through week 52 of the treatment period (*p* < 0.05), except at 09:00 on week 36 (*p* = 0.138) and week 40 (*p* = 0.402) in Cohort 3 (Fig. 1).

In the total study population, the mean ± SD change in IOP from baseline at week 52 was − 1.4 ± 2.7 mmHg (*p* < 0.001; 95% CI: –1.84 to –0.94) when IOP was measured at 09:00 (− 0.8 to − 1.9 mmHg across cohorts, all *p* < 0.05), and − 3.4 ± 3.1 mmHg (*p* < 0.001; 95% CI: –3.92 to –2.88) when IOP was measured at 11:00 (− 2.7 to − 4.1 mmHg across cohorts, all* p* < 0.001). Furthermore, among patients in Cohort 4 who did not receive any combination therapy, including ripasudil 0.4% or brimonidine 0.1%, during the screening and treatment periods (RBFC monotherapy subgroup; *n* = 21), significant and sustained IOP-lowering effects were observed (*p* < 0.05), except for IOP measurements taken at 09:00 on week 28 (*p* = 0.062) and week 32 (*p* = 0.131). In these patients, mean change in IOP from baseline at week 52 was − 2.2 ± 2.5 mmHg (*p* = 0.004; 95% CI: –3.61 to –0.86) at 09:00 and − 5.5 ± 2.5 mmHg (*p* < 0.001; 95% CI: –6.89 to –4.17) at 11:00.

Subgroup analyses showed that RBFC had stable IOP-lowering effects across patient demographics and clinical characteristics (Table 2). Throughout the treatment period, IOP was consistently reduced at 11:00 from baseline at week 8, 28, and 52 (all *p* < 0.001) in all groups stratified by age, sex, diagnosis, baseline IOP and combination therapy.

**Table 2:** Efficacy analyses for change in intraocular pressure (2 h after instillation) from baseline through week 52

	*n*	Baseline IOP^a^ (mmHg)	Change in IOP from baseline^b,c^ (mmHg)		
			Week 8	Week 28	Week 52
Total study population	179	16.8 ± 2.7	− 3.7 ± 2.5	− 3.6 ± 2.7	− 3.4 ± 3.1
Cohort					
Cohort 1	48	16.5 ± 2.0	− 3.8 ± 2.1	− 4.1 ± 2.5	− 3.5 ± 2.8
Cohort 2	44	17.5 ± 3.3	− 3.5 ± 3.1	− 3.5 ± 2.8	− 3.3 ± 3.4
Cohort 3	41	16.0 ± 2.1	− 3.2 ± 2.3	− 3.0 ± 2.5	− 2.7 ± 3.1
Cohort 4	46	17.4 ± 3.0	− 4.2 ± 2.5	− 3.6 ± 2.9	− 4.1 ± 3.0
Cohort 4 (RBFC monotherapy)^d^	21	19.0 ± 3.2	− 4.9 ± 2.0	− 4.3 ± 3.1	− 5.5 ± 2.5
Age					
< 65 years	86	16.5 ± 2.5	− 3.5 ± 2.3	− 3.8 ± 2.6	− 3.4 ± 2.9
≥ 65 years	93	17.1 ± 2.9	− 3.8 ± 2.7	− 3.3 ± 2.7	− 3.4 ± 3.3
Sex					
Male	99	16.7 ± 2.8	− 3.7 ± 2.6	− 3.5 ± 2.9	− 3.1 ± 3.3
Female	80	17.0 ± 2.7	− 3.7 ± 2.4	− 3.6 ± 2.5	− 3.9 ± 2.8
Diagnosis^e^					
POAG	151	16.5 ± 2.3	− 3.6 ± 2.4	− 3.5 ± 2.5	− 3.4 ± 3.1
OHT	26	19.1 ± 3.6	− 4.3 ± 3.4	− 3.8 ± 3.6	− 3.5 ± 3.3
Baseline IOP^a^					
< 17.5 mmHg	82	15.2 ± 1.6	− 3.4 ± 2.3	− 3.4 ± 2.3	− 3.2 ± 2.8
≥ 17.5 mmHg	97	18.2 ± 2.8	− 4.0 ± 2.7	− 3.7 ± 3.0	− 3.6 ± 3.4
Concomitant treatment with PG analogue					
Yes	134	16.6 ± 2.6	− 3.5 ± 2.5	− 3.5 ± 2.6	− 3.2 ± 3.1
No	45	17.4 ± 3.1	− 4.1 ± 2.5	− 3.6 ± 3.0	− 4.0 ± 3.0
Concomitant treatment with β-blocker					
Yes	102	16.6 ± 2.8	− 3.5 ± 2.8	− 3.3 ± 2.6	− 3.1 ± 3.2
No	77	17.2 ± 2.7	− 4.0 ± 2.2	− 3.9 ± 2.7	− 3.9 ± 3.0
Concomitant treatment with CAI					
Yes	52	15.9 ± 2.1	− 3.3 ± 2.2	− 3.2 ± 2.6	− 2.9 ± 3.1
No	127	17.2 ± 2.9	− 3.8 ± 2.7	− 3.7 ± 2.7	− 3.6 ± 3.1

β*-blocker* Beta-adrenoceptor blocker; *CAI* Carbonic anhydrase inhibitor; *EXG* Exfoliative glaucoma; *IOP* Intraocular pressure; *OHT* Ocular hypertension; *PG* Prostaglandin; *POAG* Primary open-angle glaucoma; *RBFC* Ripasudil-brimonidine fixed-dose combination; *SD* Standard deviation

Values are the number of patients or mean ± SD

^a^Baseline IOP was measured at 11:00 on day 1 of the treatment period before the initiation of RBFC

^b^IOP was measured at 11:00 (2 h after instillation) at each study visit during the treatment period

^c^In all groups, mean IOP was significantly reduced from baseline at week 8, 28, 52 (*p* < 0.001 for all time points)

^d^Subgroup of patients in Cohort 4 who did not receive any concomitant agent, including ripasudil 0.4% or brimonidine 0.1%, during the screening and treatment periods (*n* = 21)

^e^Patients with EXG were excluded due to small patient number (*n* = 2); no patients in this study had pigmentary glaucoma

### Safety of RBFC

Common AEs and ADRs reported during the treatment period (defined as events with an ADR incidence of ≥ 2%) are summarised in Table 3. In general, the incidence of common AEs and ADRs was similar across all cohorts. The majority of AEs were mild in severity, and no severe ADRs were reported.

**Table 3:** Summary of adverse events and adverse drug reactions during the treatment period

Symptoms/Signs^a^	Adverse events					Adverse drug reactions				
	Cohort 1 (*n* = 48)	Cohort 2 (*n* = 44)	Cohort 3 (*n* = 41)	Cohort 4 (*n* = 46)	Total (*n* = 179)	Cohort 1 (*n* = 48)	Cohort 2 (*n* = 44)	Cohort 3 (*n* = 41)	Cohort 4 (*n* = 46)	Total (*n* = 179)
All events	44 (91.7)	39 (88.6)	38 (92.7)	44 (95.7)	165 (92.2)	40 (83.3)	33 (75.0)	27 (65.9)	36 (78.3)	136 (76.0)
Conjunctival hyperaemia Transient^b^	33 (68.8)	22 (50.0)	21 (51.2)	28 (60.9)	104 (58.1)	33 (68.8)	22 (50.0)	21 (51.2)	28 (60.9)	104 (58.1)
	25 (52.1)	19 (43.2)	16 (39.0)	21 (45.7)	81 (45.3)	25 (52.1)	19 (43.2)	17 (41.5)	21 (45.7)	82 (45.8)
Blepharitis	14 (29.2)	11 (25.0)	9 (22.0)	12 (26.1)	46 (25.7)	8 (16.7)	9 (20.5)	8 (19.5)	6 (13.0)	31 (17.3)
Allergic conjunctivitis	9 (18.8)	8 (18.2)	11 (26.8)	10 (21.7)	38 (21.2)	8 (16.7)	8 (18.2)	8 (19.5)	9 (19.6)	33 (18.4)
Punctate keratitis	7 (14.6)	5 (11.4)	3 (7.3)	2 (4.3)	17 (9.5)	6 (12.5)	3 (6.8)	3 (7.3)	2 (4.3)	14 (7.8)
Eye irritation	6 (12.5)	2 (4.5)	2 (4.9)	3 (6.5)	13 (7.3)	6 (12.5)	2 (4.5)	2 (4.9)	3 (6.5)	13 (7.3)
Conjunctivitis	4 (8.3)	4 (9.1)	2 (4.9)	3 (6.5)	13 (7.3)	3 (6.3)	3 (6.8)	2 (4.9)	3 (6.5)	11 (6.1)
Eye pruritus	4 (8.3)	3 (6.8)	3 (7.3)	1 (2.2)	11 (6.1)	3 (6.3)	1 (2.3)	2 (4.9)	1 (2.2)	7 (3.9)
Vision blurred	3 (6.3)	1 (2.3)	0	0	4 (2.2)	3 (6.3)	1 (2.3)	0	0	4 (2.2)

*RBFC* Ripasudil-brimonidine fixed-dose combination

Values are the number of patients (%)

^a^Events listed are those with an adverse drug reaction incidence of ≥ 2%. Data are patients with ≥ 1 event; patients with multiple occurrences of the same event are counted once

^b^Transient conjunctival hyperaemia events occurred after instillation of RBFC and resolved without treatment before the next instillation

The most common AE was conjunctival hyperaemia, which was assessed to be an ADR in 104 patients (58%; 50–69% across cohorts). The majority of conjunctival hyperaemia AEs and ADRs were events that occurred after instillation of RBFC and resolved without treatment before the next instillation. Conjunctival hyperaemia ADRs were mild in severity in 100/104 patients and moderate in 4 patients.

With regards to ADRs, blepharitis was reported in 31 patients (17%; 13–21% across cohorts), allergic conjunctivitis in 33 patients (18%; 17–20% across cohorts) and conjunctivitis in 11 patients (6%; 5–7% across cohorts). Mild blepharitis, allergic conjunctivitis and conjunctivitis were reported in 28/31, 26/33 and 11/11 patients, respectively. Moderate blepharitis and allergic conjunctivitis were reported in three and seven patients, respectively.

**Fig. 2:**
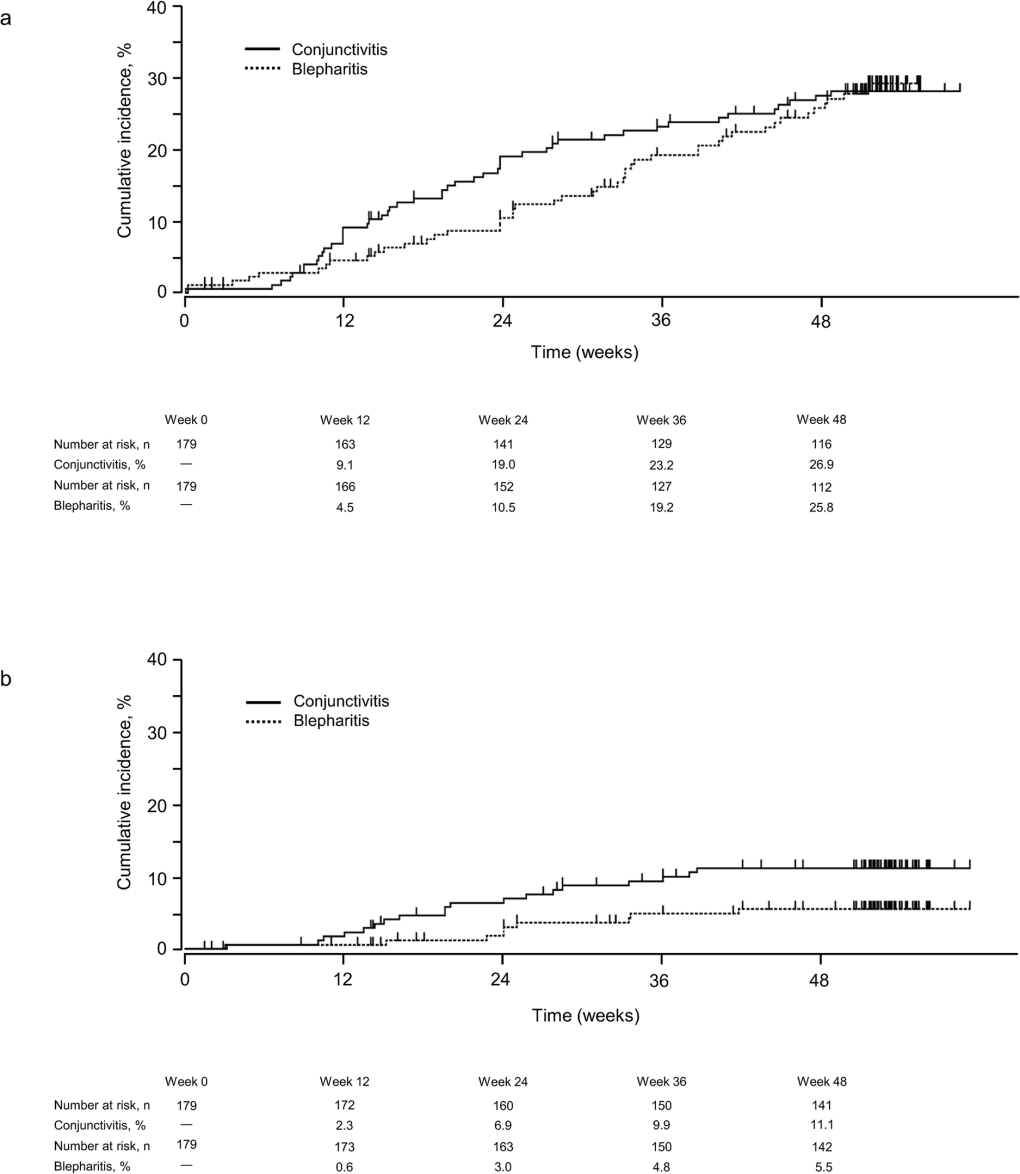
Cumulative incidence of (**a**) all blepharitis and conjunctivitis (including allergic responses) adverse events and (**b**) blepharitis and conjunctivitis events leading to study discontinuation during the treatment period

As shown in Table 4, the occurrence of AEs was analysed based on time of onset during the treatment period. The incidence of blepharitis peaked between weeks 24–36, while the incidence of allergic conjunctivitis and conjunctivitis peaked between weeks 12–24. Similarly, Kaplan–Meier analyses of the time to first onset of blepharitis and conjunctivitis (including allergic responses) indicated that the cumulative incidence of first onset increased the most between weeks 24–36 and weeks 12–24, respectively (Fig. 2a).

**Table 4:** Incidence of adverse events by time of onset during the treatment period^a^

Symptoms/Signs^b^	Total (*n* = 179)	≤ 12 weeks (*n* = 179)	> 12 to ≤ 24 weeks (*n* = 173)	> 24 to ≤ 36 weeks (*n* = 163)	> 36 to ≤ 48 weeks (*n* = 151)	> 48 weeks (*n* = 142)
Conjunctival hyperaemia	104 (58.1)	91 (50.8)	14 (8.1)	7 (4.3)	1 (0.7)	2 (1.4)
Blepharitis	46 (25.7)	8 (4.5)	10 (5.8)	14 (8.6)	11 (7.3)	4 (2.8)
Allergic conjunctivitis	38 (21.2)	12 (6.7)	15 (8.7)	8 (4.9)	7 (4.6)	2 (1.4)
Punctate keratitis	17 (9.5)	7 (3.9)	6 (3.5)	5 (3.1)	4 (2.6)	1 (0.7)
Eye irritation	13 (7.3)	13 (7.3)	0	0	0	0
Conjunctivitis	13 (7.3)	4 (2.2)	6 (3.5)	3 (1.8)	2 (1.3)	0
Eye pruritus	11 (6.1)	6 (3.4)	3 (1.7)	3 (1.8)	0	0
Vision blurred	4 (2.2)	4 (2.2)	0	0	0	0

*AE* Adverse event

Values are the number of patients (%)

^a^Data are patients with ≥ 1 AE at each treatment period; patients with multiple occurrences of the same AE in the same period are counted once. Patients with multiple occurrences of the same AE in different periods are counted once in each relevant period

^b^Events listed are those with an adverse drug reaction incidence of ≥ 2% over the 52-week treatment period

Overall, blepharitis, allergic conjunctivitis and conjunctivitis AEs that led to study discontinuation occurred in nine (5%), 16 (9%) and three (2%) patients, respectively. Kaplan–Meier analyses of conjunctivitis and blepharitis (including allergic responses) that led to study discontinuation showed that most of these events occurred from approximately week 10 onward, and the cumulative incidences of first onset increased the most between weeks 12–24 for both AEs (Fig. 2b).

There were no clinically significant changes in corneal thickness (based on ultrasound pachymetry measurements at 09:00 [before instillation] in a subset of 46 patients) or corneal endothelial cell density or morphology (based on non-contact specular microscopy measurements at 09:00 [before instillation] in all patients) from baseline. Other ocular assessments, clinical examinations and laboratory tests identified no clinically significant changes or safety signals during the treatment period.

## Discussion

This multicentre, open-label study demonstrated the 52-week IOP-lowering efficacy and safety of RBFC, both alone and in combination with current ocular IOP-lowering agents, in Japanese patients with POAG, OHT or EXG. During the treatment period, instillation of RBFC was associated with significant reductions in IOP that were maintained through 1 year. At week 52, mean reductions in IOP at 11:00 (2 h after instillation of RBFC) from baseline ranged between 2.7–4.1 mmHg among patients receiving RBFC in combination with PG analogue (Cohort 1), PG analogue and β-blocker (Cohort 2), PG analogue, β-blocker and CAI (Cohort 3) or other/no treatment (Cohort 4). RBFC also demonstrated an acceptable safety profile. The common AEs of conjunctival hyperaemia, blepharitis and allergic conjunctivitis were mostly mild in severity, and the incidences were similar between cohorts receiving different concomitant agents.

Previously, the IOP-lowering effect of RBFC was shown to be superior to that of ripasudil or brimonidine alone in patients with POAG or OHT over 8 weeks [11]. The present study treated patients for a much longer period, finding significant and stable reductions in IOP through 1 year of RBFC treatment, both alone and in combination with other IOP-lowering agents. The results of this study seem to agree with previous findings from long-term studies of ripasudil in patients with glaucoma or OHT, which demonstrated additive and stable IOP-lowering effects when ripasudil was combined with one or more concomitant agents [15, 16]. Furthermore, subgroup analyses showed that the IOP-lowering effects of RBFC were consistent across patient demographics and clinical characteristics, suggesting that it may offer an effective treatment option for a range of patients with POAG or OHT. In addition to the results of the present study, previous studies have shown ripasudil’s significant and stable IOP-lowering effects in patients with a variety of glaucoma types, including EXG, steroid-induced glaucoma, uveitis-associated glaucoma and primary angle closure glaucoma [15, 16, 18]. Taken together with the findings of previous studies, the results of this study suggest that RBFC treatment may be a useful additional therapy for glaucoma patients with various different characteristics.

There is a need for new fixed-dose combination therapies that facilitate medication adherence and improve long-term outcomes for patients with glaucoma. RBFC is the first topical fixed-dose combination treatment for glaucoma that combines a ROCK inhibitor with an α_2_-agonist. It lowers IOP via three mechanisms of action: ROCK inhibition with ripasudil increases trabecular outflow of aqueous humour, while α_2_-adrenoceptor activation with brimonidine decreases aqueous humour production and increases uveoscleral outflow [1–3]. The results of the present study demonstrate both the robust IOP-lowering effect of RBFC alone, as well as the additive efficacy of RBFC when administered with other drug classes. The similar findings between cohorts may be explained by additive IOP-lowering effects caused by the combination of triple mechanisms.

Overall, the safety and tolerability of RBFC were similar in patients receiving different combination therapies (including no treatment) for glaucoma or OHT. Conjunctival hyperaemia was the most common AE and ADR reported during the treatment period, which is consistent with previous clinical studies of ripasudil [15] and RBFC [11]. In the recent phase 3 clinical studies of RBFC in patients with POAG or OHT, the incidence of conjunctival hyperaemia ADRs was approximately 44–54% over 8 weeks of treatment [11]. In comparison, the overall incidence of conjunctival hyperaemia ADRs in the present study was 58% over 52 weeks of treatment, suggesting that the incidence of these events does not increase with long-term RBFC treatment. Moreover, the majority of conjunctival hyperaemia events in this study were mild in severity, and most were transient events that occurred immediately after instillation of RBFC and resolved without treatment. Therefore, this demonstrates that RBFC treatment does not additively increase the incidence and severity of conjunctival hyperaemia events compared with ripasudil alone.

In this study, the second- and third-most commonly reported AEs were blepharitis and allergic conjunctivitis, respectively. Blepharitis and allergic conjunctivitis events have previously been reported in patients treated with ripasudil [15, 16] and brimonidine [12–14]. Previous studies have also shown that a history of allergic reaction to other anti-glaucoma medications (including brimonidine) is a significant risk factor for the onset of blepharitis (including allergic responses) following ripasudil treatment [16, 19]. This alludes to the presence of a subpopulation that is hyper-sensitive to anti-glaucoma medications, especially brimonidine and ripasudil. Thus, it is important to assess whether the incidence and severity of these events are additively increased with combination therapy. In the present study, the 52-week incidence of blepharitis and allergic conjunctivitis ADRs was 17% and 18%, respectively, and most events were mild in severity. In comparison, the incidence of blepharitis and allergic conjunctivitis ADRs at 52 weeks were 18% and 15%, respectively, in a previous study of ripasudil [15] and ranged from 9–15% and 18–24%, respectively, in a previous study of brimonidine [12]. Together, these data suggest that RBFC treatment does not additively increase the incidence and severity of blepharitis and allergic conjunctivitis events compared with either agent alone.

When AEs in this study were assessed by time of onset, the incidence of blepharitis events was highest between weeks 24–36 and the incidence of allergic conjunctivitis events was highest between weeks 12–24. These findings are generally consistent with previous studies of ripasudil and brimonidine monotherapies [12, 14, 15]. Furthermore, the 2-year post-marketing surveillance study of ripasudil in Japanese patients with glaucoma or OHT (ROCK-J) indicated that first onset of blepharitis and conjunctivitis (including allergic response) occurred most commonly between 6–12 months after initiating ripasudil treatment and stabilised thereafter [16]. These findings collectively suggest that the risks of blepharitis and conjunctivitis AEs are highest between 12–36 weeks after initiating RBFC.

A previous study showed transient morphological changes in corneal endothelial cells a few hours after ripasudil instillation [17, 20, 21], due to the depolymerising effect of ROCK inhibition on cytoskeletal actin stress fibres [22, 23]. In the present study, corneal endothelial cell morphology, cell density and corneal thickness were monitored at each visit during the treatment period at 09:00 (before instillation of RBFC). No clinically significant changes in long-term corneal outcomes were observed.

At present, two ROCK inhibitor ophthalmic solutions are clinically available (i.e. ripasudil and netarsudil). However, these agents are structurally very different, and netarsudil is known to inhibit the norepinephrine transporter in addition to ROCK [24, 25]. As there are only short-term (4-week) trial data available to directly compare netarsudil with ripasudil [26], it is difficult to discuss the long-term efficacy and safety of these two agents. Based on previous reports, conjunctival hyperaemia occurs frequently with both drugs [26], cornea verticillata and conjunctival haemorrhage characteristically occur with netarsudil [27], and blepharitis and allergic conjunctivitis occur with ripasudil [15, 16].

The results of this study should be interpreted with caution, given its relatively small sample size of 179 patients from Japan. This small sample size, in combination with a study duration of 12 months, may have been insufficient to detect the incidence of granulomatous uveitis, a late AE associated with brimonidine treatment [28, 29]. Furthermore, ripasudil has been shown to exert anti-inflammatory effects [18, 30]; as such, combined treatment with RBFC may have masked the occurrence of this AE. Patients with a prior history of ocular surgery or laser treatment were excluded from this study, which may limit the generalisability of its findings to patients receiving RBFC in routine clinical practice. Glaucoma is a chronic eye disease that requires lifelong treatment and monitoring; therefore, longer-term studies are needed to confirm the efficacy and safety of RBFC over > 1 year of follow-up.

In conclusion, this multicentre, open-label study demonstrated the IOP-lowering efficacy and safety of RBFC in Japanese patients with glaucoma or OHT. Significant reductions in IOP were achieved with and without combination therapies and were maintained through 52 weeks, suggesting that RBFC may offer a new treatment option for the long-term management of glaucoma.

### Electronic supplementary material

Below is the link to the electronic supplementary material.

Supplementary file1 (DOCX 67.1 KB)

## Data Availability

The data from this study are not available for sharing due to patient confidentiality and ownership by Kowa Company, Ltd., Japan.

## Appendix

K-232 Clinical Study Group steering and writing committee members (i.e. authors of the current study) are as follows: Hidenobu Tanihara, MD, PhD; Tetsuya Yamamoto, MD, PhD; Makoto Aihara, MD, PhD; Noriko Koizumi, MD, PhD; Atsuki Fukushima, MD, PhD; Koji Kawakita, Satoshi Kojima, Toka Nakamura and Hideki Suganami, PhD.

K-232 Clinical Study Group study investigators are as follows: Yoshitsugu Tagawa, MD (Kitaichijo Tagawa Eye Clinic, Sapporo, Hokkaido, Japan); Hiroki Watanabe, MD (Watanabe Eye Clinic, Shiogama, Miyagi, Japan); Kiyoshi Shimizu, MD (Kawaguchiaozora Eye Clinic, Kawaguchi, Saitama, Japan); Miki Iwasaki, MD, PhD (Ryogoku Eye Clinic, Sumida, Tokyo, Japan); Sakae Matsuzaki, MD, PhD (Seijo Clinic, Setagaya, Tokyo, Japan); Hiroko Ueda, MD (Ueda Eye Clinic, Arakawa, Tokyo, Japan); Ryoko Okayama, MD and Shiro Amano, MD, PhD (Ochanomizu Inouye Eye Clinic, Chiyoda, Tokyo, Japan); Osamu Matsuoka, MD, PhD (Medical Corporation Heishinkai ToCROM Clinic, Shinjuku, Tokyo, Japan); Setsuko Hashida, MD (Hashida Eye Clinic, Shinagawa, Tokyo, Japan); Sachi Amaki Kobayashi, MD, PhD (Amaki Clinic, Minato, Tokyo, Japan); Motohiro Kiyosawa, MD, PhD (Kiyosawa Eye Clinic, Koto, Tokyo, Japan); Yuko Asai, MD (Iida Hospital, Iida, Nagano, Japan); Toru Nakajima, MD, PhD (Nakajima Eye Clinic, Fuji, Shizuoka, Japan); Yuzuru Yoshimura, MD, PhD (YOSHIMURA Eye & Internal Medical Clinic, Mishima, Shizuoka, Japan); Takao Sakai, MD, PhD (Chubu Rosai Hospital, Nagoya, Aichi, Japan); Ryoji Nomura, MD (Nomura Eye Clinic, Ichinomiya, Aichi, Japan); Satoshi Inoue, MD, PhD (Medical Corporation Heishinkai OCROM Clinic, Suita, Osaka, Japan); Ken Hayashi, MD, PhD (Hayashi Eye Hospital, Fukuoka, Fukuoka, Japan); Junko Watanabe, MD (Watanabe Eye Clinic, Shinagawa, Tokyo, Japan); Hidehito Kawabata, MD, PhD (Kawabata Eye Clinic, Urayasu, Chiba, Japan); Tomoyuki Muramatsu, MD (Muramatsu Eye Clinic, Susono, Shizuoka, Japan); Mikki Arai, MD, PhD (Arai Eye Clinic, Fukuoka, Fukuoka, Japan); and Masayoshi Migita, MD (Migita Eye Institute, Beppu, Oita, Japan).

## Declarations

### Prior presentation

This manuscript is based on work that was previously presented as an abstract at the 127th Annual Meeting of the Japanese Ophthalmological Society (6–9 April 2023; Tokyo, Japan).
