# Supplementary material for: Long-term intraocular pressure-lowering efficacy and safety of ripasudil-brimonidine fixed-dose combination for glaucoma and ocular hypertension: a multicentre, open-label, phase 3 study
Source: Graefes Arch Clin Exp Ophthalmol. 2024 Mar 2;262(8):2579–91. doi: 10.1007/s00417-024-06388-y (PMC11271374; doi:10.1007/s00417-024-06388-y)
Supplement: Supplementary file 1 — Supplementary file1 (DOCX 67.1 KB) [file 417_2024_6388_MOESM1_ESM.docx]

**Long-term intraocular pressure-lowering efficacy and safety of ripasudil-brimonidine fixed-dose combination for glaucoma and ocular hypertension: a multicentre, open-label, phase 3 study**

***Graefe's Archive for Clinical and Experimental Ophthalmology***

**Online Resource 1**

**Authors:** Hidenobu Tanihara, Tetsuya Yamamoto, Makoto Aihara, Noriko Koizumi, Atsuki Fukushima, Koji Kawakita, Satoshi Kojima, Toka Nakamura, Hideki Suganami, K-232 Clinical Study Group*

*K-232 Clinical Study Group members are listed in Appendix 1

**Corresponding author:** Hidenobu Tanihara

Department of Ophthalmology, Biei Municipal Hospital

3-8-35 Naka-machi, Biei Town, Kamikawa-gun, Hokkaido 071-0207, Japan

Email: [tanihara@pearl.ocn.ne.jp](mailto:tanihara@pearl.ocn.ne.jp)

**Supplementary Table S1** Study inclusion and exclusion criteria

| **Inclusion criteria** |
| --- |
| - Patients with POAG, OHT, EXG or pigmentary glaucoma - Aged ≥ 20 years at the time of providing consent - IOP at 9:00 on day 1 of the treatment period^a^ met the following conditions:   - IOP ≥ 15 mmHg in one eye diagnosed with POAG, OHT, EXG or pigmentary glaucoma   - IOP < 35 mmHg in both eyes |
| **Exclusion criteria** |
| - Patients who have difficulty measuring the IOP with a Goldmann applanation tonometer - Patients with narrow angle closure in either eye (Shaffer classification grade 0–2) at the start of the screening period - BCVA 20/70 or worse in either eye at the start of the screening and treatment periods - Patients with severe visual field impairment in either eye and judged by the investigator to be unsuitable for study participation - Patients with retinal disease that may cause worsening of symptoms in any eye during the study - Previous ophthalmic surgery or laser treatment in any eye before the start of the screening period, except for:   - Retinal photocoagulation or YAG laser posterior capsulotomy ≥ 90 days before the start of the screening period   - Surgery on the eyelid ≥ 120 days before the start of the screening period   - Cataract surgery ≥ 1 year before the start of the screening period - Patients for whom the following prohibited use or treatments is planned during the screening and treatment periods:   - Use of drugs to treat glaucoma or ocular hypertension (except for permitted concomitant agents specified for each cohort)   - Any ophthalmic surgery or laser treatment   - Use of contact lenses - Patients who have received intravitreal, Tenon's capsule or subconjunctival steroids in any eye ≤ 180 days before the start of the treatment period - Patients with contraindications to concomitant agent(s) specified for their assigned cohort - Patients with circulatory insufficiency such as cerebrovascular disorder, orthostatic hypotension or cardiovascular disease, and judged unsuitable to participate by the investigator - Patients with a history of hypersensitivity to ingredients of ripasudil 0.4% or brimonidine 0.1% ophthalmic solutions - Patients with a history of shock or anaphylaxis as drug hypersensitivity - Patients who are pregnant, lactating or currently seeking pregnancy - Patients who have participated in another clinical study or clinical study ≤ 30 days before the start of the screening period, or who participate in another trial at the same time as the present study - Patients who have previously received RBFC eye drops at any time - Patients deemed otherwise unsuitable for study participation by the investigator |

*BCVA* Best-corrected visual acuity, *EXG* Exfoliative glaucoma, *IOP* Intraocular pressure, *OHT* Ocular hypertension, *POAG* Primary open-angle glaucoma, *RBFC* Ripasudil-brimonidine fixed-dose combination, *YAG* Yttrium aluminium garnet.

^a^IOP used to determine eligibility was measured before the initiation of RBFC and based on the median of 2–3 readings for each eye.

**
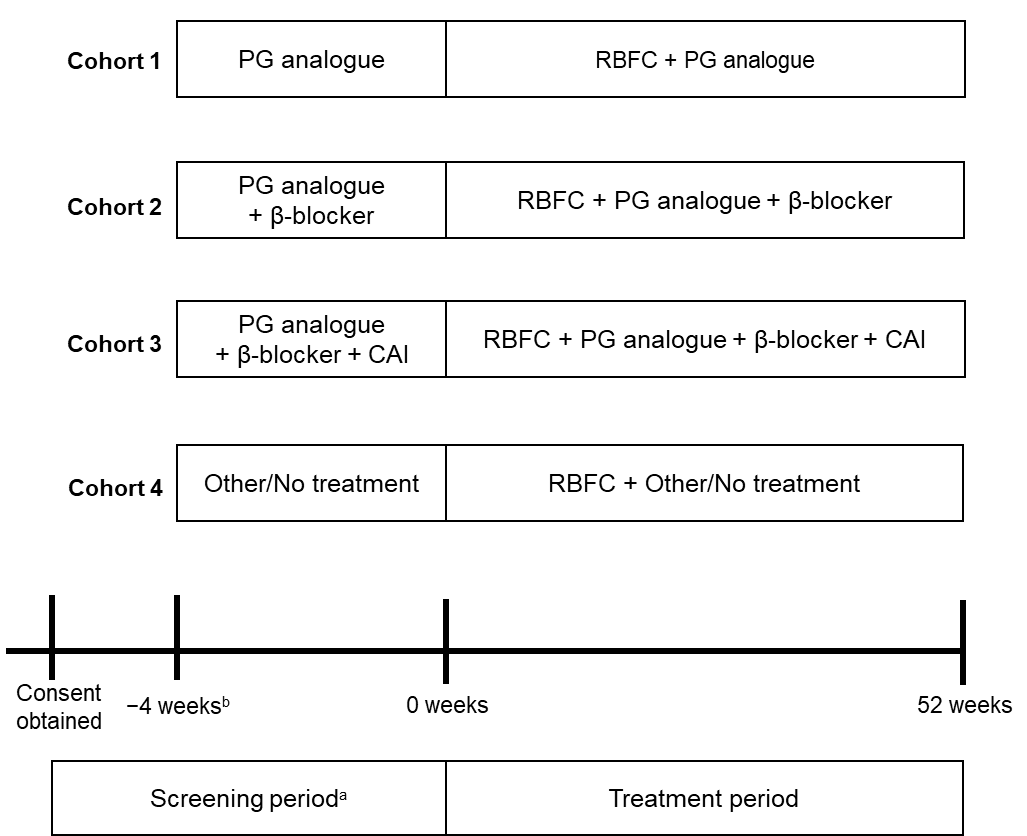
**

**Supplementary Fig. S1** Study design

β*-blocker* Beta-adrenoceptor blocker, *CAI* Carbonic anhydrase inhibitor, *PG* Prostaglandin, *RBFC* Ripasudil-brimonidine fixed-dose combination

^a^During the screening period, patients in all cohorts could receive ripasudil 0.4% or brimonidine 0.1% per the investigator’s discretion; these agents were to be administered for ≥ 4 weeks, up to a maximum of 6 weeks

^b^In Cohorts 1–3, the duration of the screening period was ≥ 4 weeks, up to a maximum of 6 weeks. For patients in Cohort 4 who did not receive any combination therapy, including ripasudil 0.4% or brimonidine 0.1%, during the screening period, the treatment period could start ≥ 1 day after the start of the screening period, provided that any applicable washout criteria were met

**
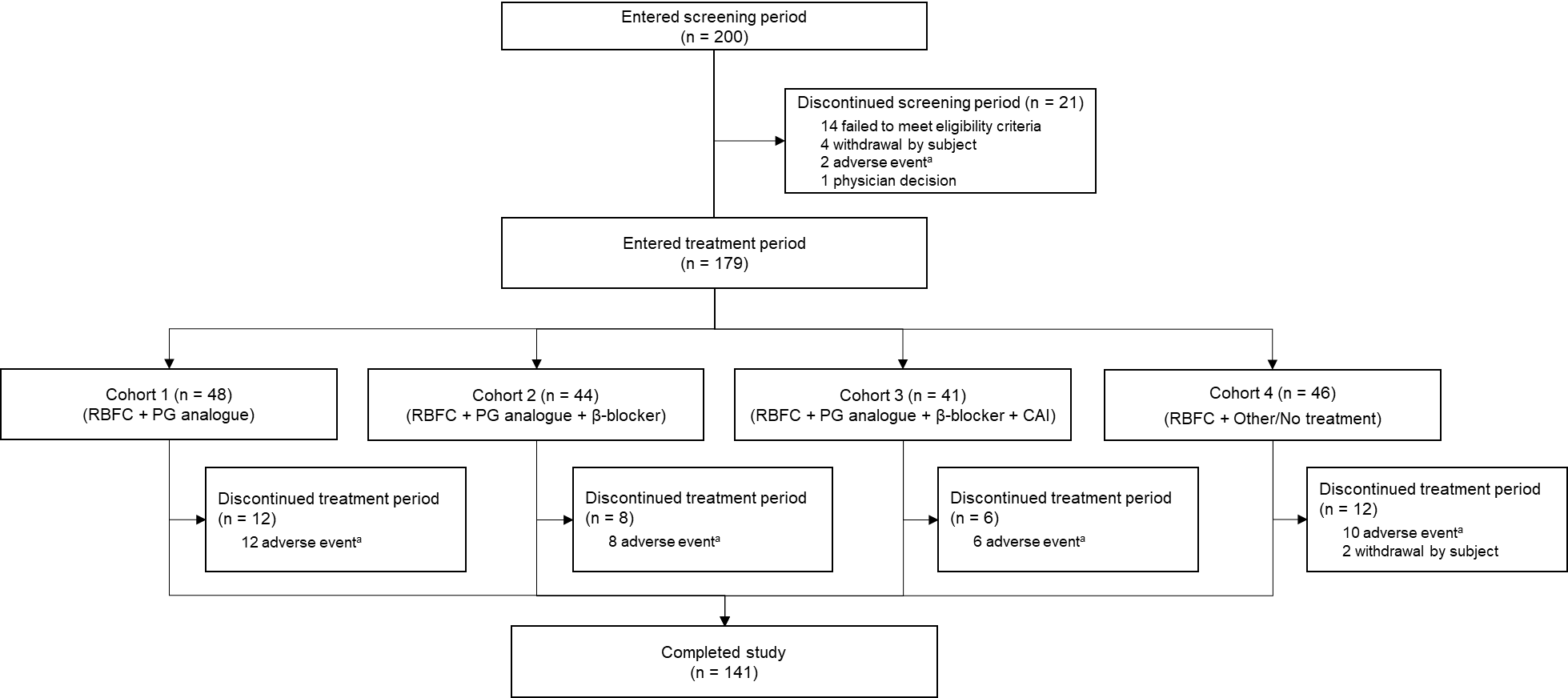
Supplementary Fig. S2** Patient flow diagram

β*-blocker* Beta-adrenoceptor blocker, *CAI* Carbonic anhydrase inhibitor, *PG* Prostaglandin, *RBFC* Ripasudil-brimonidine fixed-dose combination
